# Development of gold nanoparticles biosensor for ultrasensitive diagnosis of foot and mouth disease virus

**DOI:** 10.1186/s12951-018-0374-x

**Published:** 2018-05-11

**Authors:** Mervat E. Hamdy, Michele Del Carlo, Hussein A. Hussein, Taher A. Salah, Ayman H. El-Deeb, Mohamed M. Emara, Guilia Pezzoni, Dario Compagnone

**Affiliations:** 1 Department of Biotechnology Animal Health Research Institute, Agriculture Research Centre, Giza, 12618 Egypt; 2 Department of Biosciences and Technology for Food, Agriculture and Environment, University of Teramo, 64023 Teramo, Italy; 3 Department of Virology, Faculty of Veterinary Medicine, Cairo University, Giza, 12211 Egypt; 4 Nanotechnology Research Centre, British University, Cairo, Egypt; 5 Nanotechnology and Advanced Materials Central Lab, Agricultural Research Centre, Giza, Egypt; 6 Department of Biotechnology, Zooprofilattico Institute of Lombardy and Emilia Romagna (IZSLER), 25124 Brescia, Italy

**Keywords:** AuNPs-FMDV biosensor, Diagnosis, rRT-PCR

## Abstract

**Background:**

Nano-PCR is a recent tool that is used in viral diseases diagnosis. The technique depends on the fundamental effects of gold nanoparticles (AuNPs) and is considered a very effective and sensitive tool in the diagnosis of different diseases including viral diseases. Although several techniques are currently available to diagnose foot and mouth disease virus (FMDV), a highly sensitive, highly specific technique is needed for specific diagnosis of the disease. In the present work, a novel AuNPs biosensor has been designed using thiol-linked oligonucleotides that recognize the conserved 3D gene of FMDV.

**Results:**

The AuNPs-FMDV biosensor specifically recognizes RNA standards of FMDV, but not that of swine vesicular disease virus (SVDV) isolates. The analytical sensitivity of the AuNPs-FMDV biosensor was 10 copy number RNA standards in RT-PCR and 1 copy number RNA standard in real-time rRT-PCR with a 94.5% efficiency, 0.989 R^2^, a − 3.544 slope and 100% specificity (no cross-reactivity with SVDV). These findings were confirmed by the specific and sensitive recognition of 31 Egyptian FMDV clinical isolates that represents the three FMDV serotypes (O, A, and SAT2).

**Conclusions:**

The AuNPs-FMDV biosensor presents in this study demonstrates a superior analytical and clinical performance for FMDV diagnosis. In addition, this biosensor has a simple workflow and accelerates epidemiological surveillance, hence, it is qualified as an efficient FMDV diagnosis tool for quarantine stations and farms particularly in FMDV endemic areas.

**Electronic supplementary material:**

The online version of this article (10.1186/s12951-018-0374-x) contains supplementary material, which is available to authorized users.

## Background

PCR enhancers include small organic molecules (dimethyl sulphoxide, glycerol, betaine monohydrate, and formamide) [1], non-ionic detergents (0.1–1% Triton X-100, and Tween-20), proteins, such as bovine serum albumin (BSA), and single-stranded DNA binding proteins (SSBPs) [2] had been extensively used to improve PCR application for routine diagnostic purposes. However, there is still a need to develop more appropriate additives to enhance the specificity and efficiency of PCR, which is considered a great challenge [3]. In recent years, huge research efforts have been directed to using various nanoparticle (NP)-based enhancers, that lead to the development of what so called nanomaterials-assisted PCR (nano-PCR) [4, 5]. NP-based enhancers such as gold nanoparticles (AuNPs) [6], semiconductor quantum dots [7], carbon nanotubes [8] and carbon nano powders [9], have shown to improve PCR specificity and efficiency by different working mechanisms that range from relieving the secondary DNA structure in GC-rich regions or in long amplification products, the reduction of the template melting temperature, the stabilization of DNA polymerases and the enhancement of its activity, to the prevention of adsorption of polymerases to plastic ware [10].

Out of these enhancers, AuNPs stands out as the most well-known and effective enhancer that is capable to improve two PCR rounds with respect to both yield and specificity [11]. In the presence of the appropriate amount of AuNPs, the target product may be achieved after only six PCR cycles [12]. In line with this, AuNPs have been reported to increase the sensitivity of PCR detection five- to tenfold in a conventional PCR system and 10^4^-fold in a real-time PCR system [13]. Moreover, AuNPs modulate the activity of DNA polymerases and achieve hot-start activity in the presence of conventional *Pyrococcus furiosus* (Pfu) polymerases [14]. Besides, AuNPs have unique chemical and physical properties such as design flexibility, large surface-to-volume ratio, simple surface modification with multivalent ligands, catalytic effect for electrochemical reactions, improvement of electron transfer, and labelling of biomolecules, which make such enhancer particularly appropriate for designing new and improved biosensors [15, 16]. Indeed, AuNPs had been used as immunosensor [16], DNA sensors [17], streptavidin-AuNPs [18], and the layer-by-layer amino-thiophenol-AuNPs network [19].

AuNPs have been also used in RNA detection where a rapid label-free visual assay has been developed using peptide nucleic acid (PNA) probes and AuNPs. The specific agglomerative behaviour of PNA with AuNPs can detect as low as 5–10 ng of viral RNA from various biological samples, indicating the sensitivity of this assay [20]. Hence, it was extensively used in viral diagnosis. A rapid and specific diagnosis of Japanese encephalitis virus (JEV) was achieved using an AuNPs-based RT-PCR and rRT-PCR assay [21]. In addition, a specific label-free AuNPs immunosensor was optimized and applied in the diagnosis of dengue virus using [22], layer-by-layer AuNPs hybridization on a quartz crystal microbalance (QCM) DNA sensing system [23] and 4G2 antibody-AuNPs surface enhanced Raman spectroscopy (SERS) fingerprinting [24]. Moreover, an AuNPs-immunochromatographic assay (AuNPs-ICA) was used for detecting severe fever with thrombocytopenia syndrome virus (SFTSV) infection with a sensitivity of 98.4% for IgM and 96.7% for IgG [25]. This significant role of AuNPs in viral diagnosis was also highlighted in the colorimetric detection of influenza virus using AuNPs-RT loop-mediated isothermal amplification (AuNPs-RT-LAMP) that targets the virus M protein gene and showed 100% specificity and 98.6% sensitivity in comparison to conventional RT-LAMP [26]. In addition, the influenza virus was detected using a portable AuNPs biosensor and based on surface-enhanced Raman scattering (SERS) with a 1 pg/µL detection limit [27, 28]. Influenza virus was detected also by biosensors based on dynamic light scattering (DLS) [29].

Foot and Mouth Disease (FMD) is one of the most infectious viral diseases with the potential for devastating economic, social and environmental impacts. The aetiological agent (FMDV) belongs to the Aphthovirus genus and family Picornaviridae and is present as seven serotypes (A, O, C, Asia1, and SAT 1, 2, and 3) with multiple antigenic and genetic variants. Currently, the reference method for the detection of all FMDV serotypes is real-time RT-PCR, which is based on protocols generated from Callahan et al. [30] and Reid et al. [31] that detect the virus RNA-dependent RNA polymerase (3D gene) and the 5′ untranslated region (5′UTR), respectively. Although, the two methods had been used extensively used for the virus detection and in-turn the disease diagnosis, they need further optimization. While there are several available methods to diagnose FMDV such as virus neutralization test, ELISA, and RT-PCR, there is still an essential need to improve these methods to make it more sensitive and specific.

In the present study, we evaluated AuNPs-based rRT-PCR for the detection of foot and mouth disease virus (FMDV). We found that AuNPs-FMDV biosensor was designed using thiol-linked primers of the 3D rRT-PCR [30]. This biosensor has been validated with the FMDV RNA standard from the synthetic 3D gene of FMDV. Application of the AuNPs-FMDV biosensor in RT-PCR and rRT-PCR was conducted to test the enhancement effect of the AuNPs-FMDV biosensor in the specificity, the analytical sensitivity, dynamic range, efficiency and the limit of detection (LOD) of RT-PCR and rRT-PCR.

## Methods

### Synthesis and characterization of colloidal AuNPs (13 nm)

The synthesis of the AuNPs was conducted as previously described [32] and characterized using high resolution Transmission Electron Microscopy (TEM) (to examine the shape and size of AuNPs), UV–Vis spectrophotometer (to show absorption peaks of AuNPs), Dynamic light scattering (DLS) (to determine the size of the AuNPs using Zetasizer), and zeta potential distribution (to determine the net charge of the AuNPs using the Zetasizer).

### Design and characterization of AuNPs-FMDV biosensor

To ensure the maximum thiol-linked oligonucleotide loading density on the AuNPs surface, the 13 nm AuNPs were synthetized as previously reported [33]. The 3D specific primers described by Callahan et al. [30] were modified by the addition of the poly(A) spacer and thiol linkers to enable conjugation of AuNPs. The thiol-linked poly(A) oligonucleotides length was 22 bases for the forward primer for the 3D gene and 17 bases for the reverse primer reported in Table 1. This length has been reported to ensure optimum immobilization [34], whereas the use of poly(A) nucleotides were used as spacers to organize the immobilization [33]. Thiol-linked oligonucleotides were deprotected by a disulphide cleavage buffer and desalted and purified using the Nap-5 column as previously reported [35]. The conjugation process of AuNPs and the functionalization of AuNPs with poly(A) thiol-linked oligonucleotides were conducted following the published protocol [35] with slight modification; where a 400 nM concentration of poly(A) thiol-linked oligonucleotides were used for 0.7 mL of AuNPs. The AuNPs-FMDV biosensor was characterized as mentioned above (characterization of colloidal AuNPs).

**Table 1:** Sequences of the 3D poly A thiol-linked oligonucleotides

No.	Name	Modification and sequence
1	Forward primer	(5′ Thiol group-AAAAAAAAAA-ACTGGG TTT TAC AAA CCT GTG A 3′)
2	Reverse primer	(5′ Thiol group-AAAAAAAAAA-GCG AGT CCT GCC ACG GA 3′)
3	TaqMan^®^ probe	FAM (5′ TCCTT TGCAC GCCGT GGGAC 3′)

### Real time RT-PCR

FMDV rRT-PCR was done using QuantiTect Kit (Qiagen) in a real-time PCR machine (StepOne, Applied Biosystems) with a thermal profile according to the manufacturer’s instructions. To study the effect of different thiol-linked oligonucleotides concentrations (400, 600, and 800 nM) during AuNPs-FMDV biosensor design a standard curve of rRT-PCR for each concentration was generated using an RNA standard (GeneArt, Thermo Fisher). For studying the effect of rRT-PCR conditions (salt concentration in PCR buffer and denaturation temperature) on the AuNPs-FMDV biosensor, PCR products were characterized with the UV–Vis–NIR spectrophotometer to compare the optical density of the AuNPs-FMDV biosensor before and after the rRT-PCR.

### Validation and harmonization of the analytical and diagnostic sensitivity for the AuNPs-FMDV biosensor with rRT-PCR and RT-PCR

The AuNPs-FMDV biosensor was evaluated for the analytical sensitivity using the FMDV 3D gene synthesized by (GeneArt, Thermo Fisher). Additionally, the AuNPs-FMDV biosensor was evaluated for the diagnostic sensitivity using the previously sequenced FMDV isolates (A Iran 05, O1 Manisa, and SAT2 Gharbia). And swine vesicular disease virus (SVDV), which were used as a negative control to evaluate the optimal AuNPs oligonucleotides and probe concentrations. RNA extraction was conducted using a QIAamp viral RNA mini kit (Qiagen) according to the manufacturer’s instructions and one-step rRT-PCR was done as mentioned above.

### Application of the AuNPs-FMDV biosensor with clinical samples

Thirty-one clinical field samples (unruptured and recently ruptured vesicles in the buccal cavity, vesicular fluid, epithelium and hearts) were collected from cattle, buffalos, and calves from Egypt from March 2012 to September 2015 and were tested with the FMDV-AuNPs biosensor using rRT-PCR technique.

## Results

### Design and characterization of AuNPs-FMDV biosensor

**Fig. 1:**
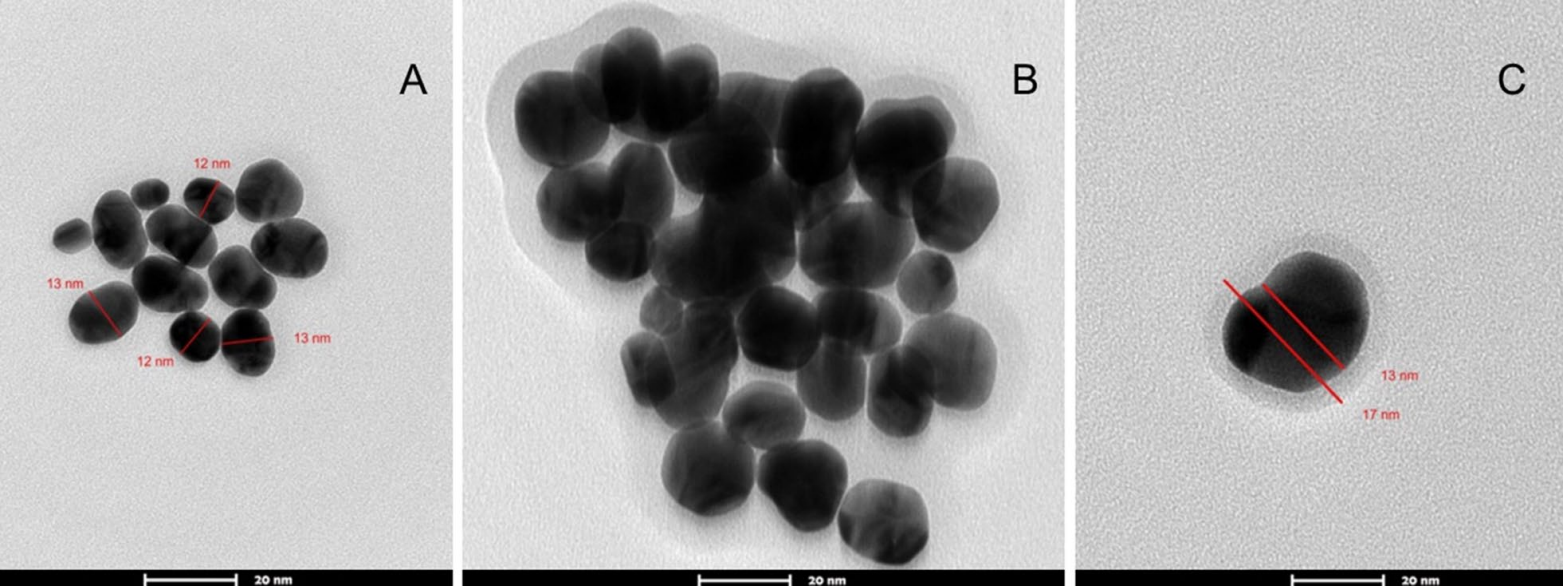
High resolution transmission electron microscope of the naked AuNPs and AuNPs-FMDV biosensor: **A** the electron microscopy image showing 13 nm naked AuNPs. **B**, **C** The electron microscopy image showing slight increase in AuNPs size 17–20 nm with grey conjugation zone and forming of AuNPs-FMDV biosensor

**Fig. 2:**
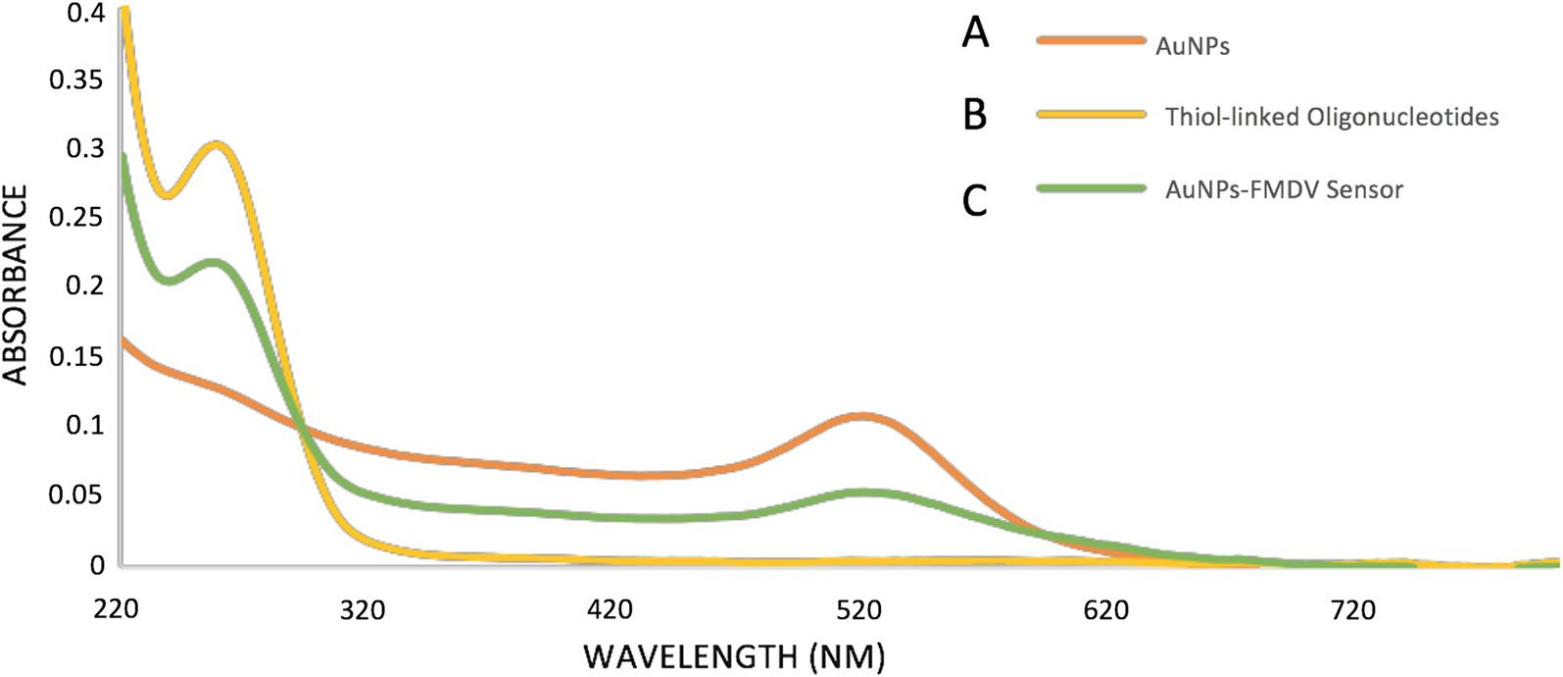
UV–Vis–NIR spectrophotometer of the naked AuNPs and AuNPs-FMDV biosensor: curve **A** the UV–Vis–NIR spectrophotometer in the visible region image showing one absorption peak of 520 nm representing 13 nm of naked AuNPs. Curve **B** the UV–Vis–NIR spectrophotometer in the visible region image showing one absorption peak of 260 nm representing free oligonucleotides. Curve **C** the UV–Vis–NIR spectrophotometer in the visible region image showing two absorption peaks one for AuNPs at 520 nm and the other was for conjugated oligonucleotides at 260 nm forming of AuNPs-FMDV biosensor

**Fig. 3:**
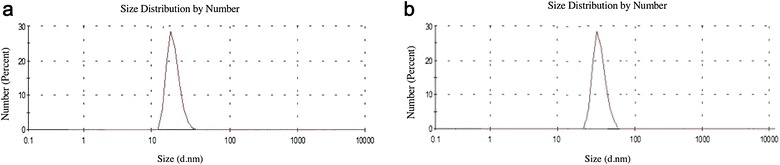
The dynamic light scattering of the naked AuNPs and AuNPs-FMDV biosensor: curve **a** demonstrates a peak of size distribution by number representing 13 nm of naked AuNPs. Curve **b** demonstrates a peak of size distribution by number showing slight increase in size due to the conjugation process representing 17–20 nm AuNPs-FMDV biosensor

**Fig. 4:**
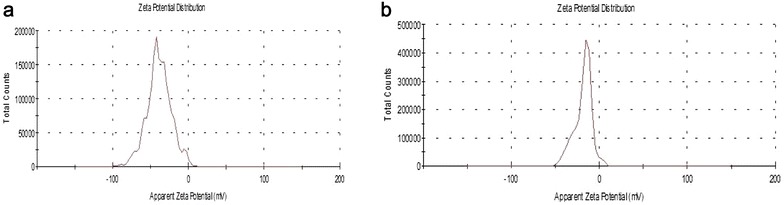
The zeta potential of the naked AuNPs and AuNPs-FMDV biosensor: curve **a** illustrates a peak of zeta potential distribution representing surface charges (− 38.9 m V) of naked AuNPs. Curve **b** illustrates a peak of zeta potential distribution representing slight decrease in AuNPs surface charge (from − 38.9 to − 18.3 m V) due to the conjugation process and forming of AuNPs-FMDV biosensor (**b**)

Previous studies showed that synthesized colloidal AuNPs could be used as high performance biosensors in many applications in vitro and in vivo. One of these applications is the detection of viral pathogens using AuNPs biosensors, which offered a significant improvement in the field of viral diseases diagnosis. Therefore, we sought to design an AuNPs biosensor for FMDV detection in a way to enhance the diagnostic methods for the disease. This process is based on two major steps, the first step is to synthesize and characterize colloidal AuNPs and the second is to adapt it to specifically detect FMDV. The colloidal AuNPs were synthesized as described in “Methods” to produce particles of 13 nm in diameter, which are coated with citrate. Citrate capping layers were synthesized to protect the AuNPs to be highly stable and can be stored under sterile conditions for several months. As shown in the TEM images in Fig. 1A, we could successfully synthesize colloidal AuNPs particles with a uniform shape and size (12–13 nm in diameter). The size of the particles was further confirmed by UV–Vis spectrum analysis that revealed a peak at absorbance–wavelength of 520 nm, which represents the 13 nm diameter AuNPs particles (Fig. 2A). In line with this, DLS data showed a specific peak of the synthesized AuNPs that corresponds to a diameter size of 13 nm (Fig. 3a). Finally, the characteristics of the synthesized AuNPs, were further confirmed by zeta potential distribution with a peak of − 38.9 m V, surface charge and a typical AuNPs distribution curve (Fig. 4a). Altogether, these results confirm the successful production of a stable citrated protected colloidal AuNPs particles that can be stored under sterile conditions for several months and used in further experiments to establish AuNPs-FMDV biosensor.

To generate AuNPs-FMDV biosensor, thiol-linked poly(A) oligonucleotides that recognize FMDV 3D gene was conjugated to the colloidal AuNPs as described in “Methods” and the conjugation of the oligonucleotides to AuNPs was monitored by different characterization methods. TEM images of the conjugated AuNPs showed particles that are slightly bigger in size (17–20 nm diameter) than the unconjugated (naked) 13 nm in diameter AuNPs (compare Fig. 1A–C. Moreover, a light grey zone appears to surround each AuNPs conjugated particle (Fig. 1B, C), whereas this zone was not observed in naked AuNPs (Fig. 1A), suggesting that this may be the area representing the conjugation zone. To further confirm the conjugation process, the UV–Vis–NIR spectrophotometer absorbance of the conjugated AuNPs as well as thiol-linked poly(A) oligonucleotides was compared to that of the naked AuNPs. As shown in Fig. 2A, naked AuNPs showed a peak at absorbance–wavelength of 520 nm, whereas an absorbance peak of 260 nm was observed with thiol oligonucleotides alone (Fig. 2B). In contrast, the combination of AuNPs and the thiol oligonucleotides (conjugated AuNPs) showed two absorption peaks, one for the AuNPs at 520 nm and the other for conjugated thiol-linked poly(A) oligonucleotides at 260 nm (Fig. 2C), indicating the conjugation of AuNPs-FMDV oligonucleotides via thiol linkage. Moreover, DLS analysis confirmed such conjugation, where the addition of FMDV oligonucleotides shifted the naked AuNPs peak from 13 to 17–20 nm (compare Fig. 3a, b). In a final attempt to fully characterize the produced AuNPs-FMDV oligonucleotides conjugation, zeta potential of conjugated AuNPs particles was measured and compared to that of the naked AuNPs. As shown in Fig. 4 the conjugation of FMDV oligonucleotides to naked AuNPs induces a pronounced change in the zeta potential measurements from − 38.9 (naked AuNPs, Fig. 4a) to − 18.3 m V (AuNPs-FMDV, Fig. 4b). Collectively these findings ensure that we could produce AuNPs conjugated to FMDV oligonucleotides that can be used as a possible biosensor for FMDV detection.

### Validation and harmonization of the analytical and diagnostic sensitivity for the AuNPs-FMDV biosensor with rRT-PCR and RT-PCR

**Fig. 5:**
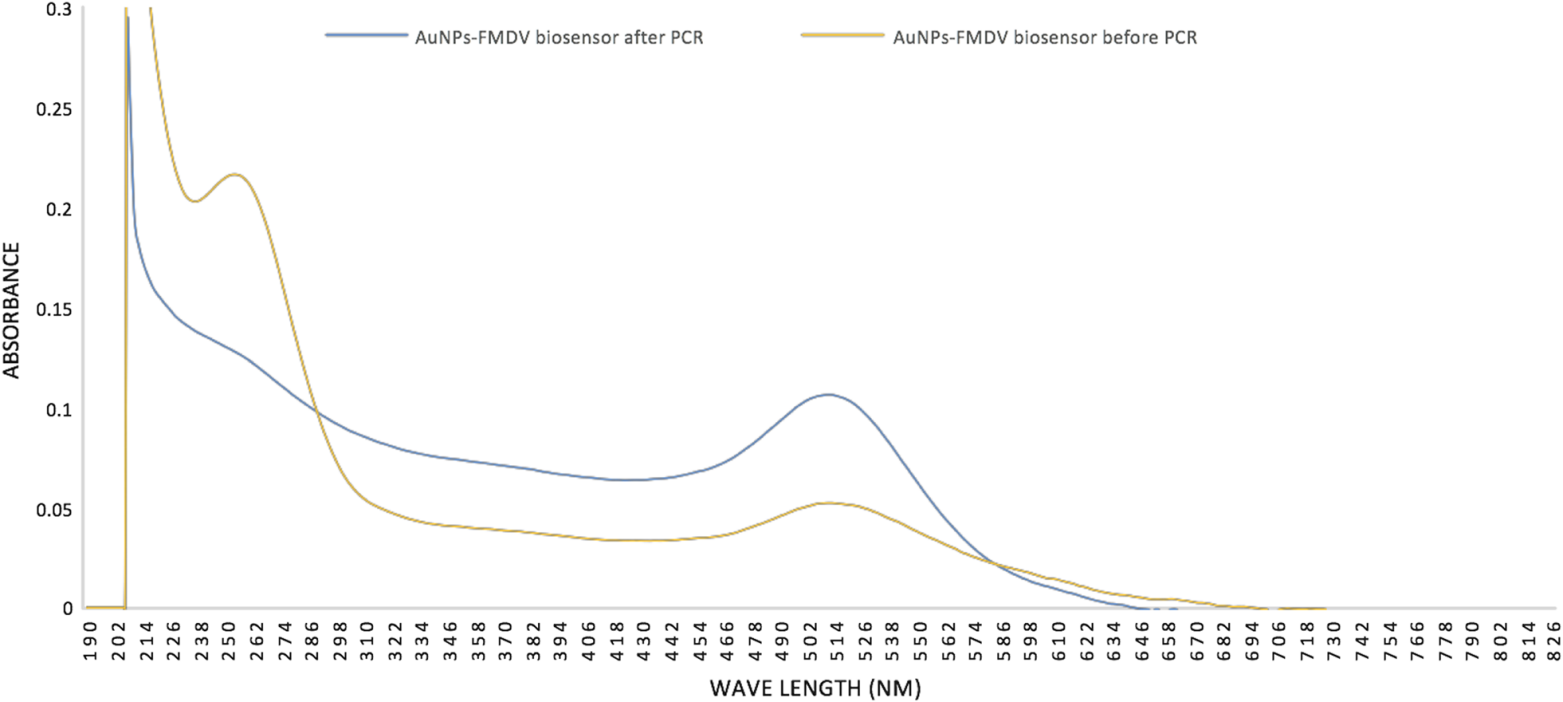
The optical density of AuNPs-FMDV biosensor before and after PCR using UV–Vis spectrum for validation of AuNPs-FMDV biosensor PCR condition: The peaks of absorbance–wavelength of AuNPs after PCR (blue curve) have little variety from the peaks of absorbance–wavelength of AuNPs before PCR (yellow curve), both have the same peak at 520 nm of absorbance–wavelength with slight disappearance of immobilized primer peak (260 nm) due to consuming of primers in PCR reaction and AuNPs peak not affected before or after PCR

To test the predictive AuNPs-FMDV biosensor to be used in diagnosis, we have first to optimize it and validate its activity under the known PCR conditions (salt concentration in the PCR buffer and denaturation temperature). The optical densities of the AuNPs-FMDV biosensor before and after the PCR was analysed using UV–Vis spectrophotometer absorbance. A defined peak at 520 nm of the absorbance–wavelength was observed before and after PCR reaction indicating the stability of AuNPs during the reaction. However, there was a slight expected disappearance of the immobilized thiol-linked oligonucleotides peak at 260 nm, which might be due to primer consumption during PCR reaction (Fig. 5).

Next, we tested the optimum thiol-linked oligonucleotide concentration(s) (400, 600 and 800 nM) on the AuNPs-FMDV biosensor using rRT-PCR, where a standard curve was generated using serially diluted standard FMD RNA to determine the limit of detection (LOD). The LOD, slope, and efficiency percentage illustrated in Table 2 and Fig. 7a, b showed that the thiol-linked oligonucleotide concentration of 400 nM has the most acceptable parameters with detection limit as low as 1 copy number, a slope − 3.544, and an efficiency of 94.5%. Conversely, the thiol-linked oligonucleotide concentration of 600 and 800 nM had poor detection (LOD of 100 copy numbers), poor efficiencies of 120 and 346%, and a slope of − 2.8 and − 1.53; respectively (Additional file 1: Fig. S2, Additional file 3: Fig. S3). Based on this data, we continued our experiments using 400 nM thiol-linked oligonucleotide concentration.

**Table 2:** Results of analytical sensitivity, dynamic range and limit of detection (LOD) with different conditions

No.	AuNPs 0.7 nM-FMDV sensor primer Concentration	Efficiency %	Slope	LOD
1	400 nM primer	94.5	− 3.544	1 copy
2	600 nM primer	120	− 2.8	100 copies
3	800 nM primer	346	− 1.53	100 copies

### Validation of the AuNPs 3D FMDV biosensor specificity

To exclude the possibility of cross reactivity diagnosis, the AuNPs-FMDV biosensor has been validated for specificity using RNA standard of 10 the FMDV closely related family member [swine vesicular disease virus isolates (SVDV)]. In parallel, serial dilution of the standard RNA for the FMDV 3D gene from Log 5–1 copy number was used as a positive control. Using conventional PCR or rRT-PCR reactions, we were not able to detect any specific bands or signals with SVDV isolates, respectively. In contrast, the AuNPs-FMDV biosensor specifically detected FMDV RNA as clearly shown by the very specific PCR bands representing 3D gene. (Additional file 1: Fig. S1). Moreover, PCR reaction could detect greater than Log 3 copy numbers with observed non-specific bands. Interestingly, rRT-PCR data showed an increase in the sensitivity up to tenfold indicating that the AuNPs-FMDV biosensor could detect greater than 100 copy numbers in the RT-PCR. This data, confirm that AuNPs-FMDV biosensor is a highly sensitive method that specifically detect FMDV without any cross reactivity with other related family members.

### Application of the AuNPs-FMDV biosensor to detect FMDV in clinical samples

To test the developed AuNPs-FMDV biosensor for FMDV diagnosis, the presence of FMDV RNA in clinical isolates were tested using conventional rRT-PCR and AuNPs-FMDV biosensor rRT-PCR. Thirty-one clinical samples were collected from cattle, buffalos, and calves that have FMDV symptoms from different Egypt governorates over the period of 2 years (March 2012 to September 2015). Samples were processed, RNA was isolated, and rRT-PCR was performed as described in “Methods”. To ensure the reliability of our comparison, samples were done in triplicates and the Ct values were determined at the same threshold value. Absolute Ct values were used to analyze the difference in detection levels of FMDV RNA between the two rRT-PCR methods. In all the 31 samples tested, a significant enhancement in FMDV RNA detection was observed in AuNPs-FMDV biosensor rRT-PCR as compared to the conventional rRT-PCR. Ct values of were 4.09 (lowest) and 27.04 (highest) were detected by AuNPs-FMDV biosensor, whereas the lowest and the highest values of the conventional rRT-PCR were 7.03 and 29.82, respectively (Fig. 8; Table 3). Moreover, different C_t_ values were detected across all the sample pools tested, however, the enhancement difference range (at least three cycles enhancement) between AuNPs-FMDV biosensor rRT-PCR and the conventional rRT-PCR remains the same. Importantly, AuNPs-FMDV biosensor differentially diagnosed FMDV serotypes (O, A, and SAT2). This data show that AuNPs-FMDV biosensor is a sensitive and specific method for the detection of different FMDV serotypes.

**Table 3:** Results of clinical samples with classical rRT-PCR reagents and with AuNPs-FMDV biosensor

No.	Serotype	Source	Type of sample	rRT-PCR results	
				Classical reagents (C)	AuNPs-FMDV biosensor (S)
*1*	*O*	*Cattle-Menoufia—2014*	*Vesicular fluid*	*17.3*	*15.6*
*2*	*O*	*Cattle-Beheira—2014*	*Epithelium*	*21.8*	*18.4*
*3*	*O*	*Cattle-Sharqia—2014*	*Epithelium*	*27.8*	*25.7*
*4*	*O*	*Buffalo-Kafr El-Sheikh—2014*	*Epithelium*	*18.32*	*15.2*
*5*	*O*	*Cattle-Damietta—2013*	*Tongue*	*26.99*	*24.44*
*6*	*O*	*Cattle-Damietta—2013*	*Epithelium*	*14.1*	*12.29*
*7*	*O*	*Cattle-Damietta—2013*	*Vesicular fluid*	*17.29*	*13.54*
*8*	*O*	*Cattle-Beheira—2014*	*Epithelium*	*29.66*	*26.48*
*9*	*O*	*Cattle-Beheira—2014*	*Epithelium*	*29.82*	*27.44*
*10*	*O*	*Buffalo-Kafr El-Sheikh—2014*	*Epithelium*	*19.32*	*16.26*
*11*	*O*	*Cattle-Menoufia—2014*	*Vesicular fluid*	*24.03*	*21.02*
*12*	*O*	*Cattle-Beheira—2013*	*Heart*	*17.6*	*15.89*
*13*	*O*	*Cattle-Beheira—2013*	*Epithelium*	*26.82*	*24.74*
***14***	***A***	***Cattle-Giza—2012***	***Vesicular fluid***	***7.03***	***4.09***
***15***	***A***	***Cattle-Giza—2012***	***Vesicular fluid***	***15.9***	***12.93***
***16***	***A***	***Cattle-Beni Suef—2014***	***Epithelium***	***20.7***	***17.31***
***17***	***A***	***Cattle-Beni Suef—2014***	***Epithelium***	***19.6***	***16.41***
***18***	***A***	***Cattle-Sharqia—2013***	***Vesicular fluid***	***25.03***	***22.08***
***19***	***A***	***Cattle-Sharqia—2013***	***Epithelium***	***15.87***	***12.72***
***20***	***A***	***Cattle-Sharqia—2013***	***Vesicular fluid***	***17.3***	***14.84***
***21***	***A***	***Cattle-Menoufia—2013***	***Epithelium***	***9.10***	***6.23***
***22***	***A***	***Cattle-Qena—2013***	***Epithelium***	***14.65***	***11.74***
**23**	**SAT2**	**Cattle-Dakahleya—2012**	**Heart**	**7.03**	**4.09**
**24**	**SAT2**	**Buffalo-Damietta—2012**	**Vesicular fluid**	**15.9**	**12.93**
**25**	**SAT2**	**Cattle-Qena—2014**	**Epithelium**	**20.7**	**17.31**
**26**	**SAT2**	**Cattle-Alexandria2012**	**Epithelium**	**27.93**	**24.35**
**27**	**SAT2**	**Cattle-Dakahleya—2012**	**Heart**	**17.28**	**13.86**
**28**	**SAT2**	**Cattle-Qena—2014**	**Epithelium**	**7.03**	**4.09**
**29**	**SAT2**	**Cattle-Menoufia—2014**	**Epithelium**	**21.85**	**18.42**
**30**	**SAT2**	**Cattle-Menoufia—2014**	**Epithelium**	**12.63**	**11.47**
**31**	**SAT2**	**Cattle-Menoufia—2014**	**Vesicular fluid**	**9.55**	**6.85**

## Discussion

The mass culling of animals has generated interest in the development of sensitive diagnostic techniques and safe effective vaccines to confine outbreaks [36, 37], especially for FMDV, as it is highly variable, and its serotyping needs nucleotide sequencing and continuous monitoring of the primers’ sensitivity and specificity [38]. The integration among science branches can develop new sensitive diagnostic techniques and increase the sensitivity, specificity and the efficiency of current diagnostic techniques. In the present study, an AuNPs-FMDV biosensor was designed. To obtain this biosensor, naked AuNPs were synthesized using a citrate reduction method [32]. Characterization of the prepared particles was conducted by 4 techniques, which demonstrate an average diameter of 13 nm under transmission electron microscopy (TEM) (Fig. 1a), and UV–Vis spectrum analysis demonstrated the specific peak of the AuNPs at the absorption wavelength of 520 nm (Fig. 2a) and dynamic light scattering of 13 nm AuNPs (Fig. 3a). Moreover, the net charges of the AuNPs were characterized by the Zeta sizer, which showed a peak of zeta potential distribution representing the surface charges (− 38.9 m V) of the naked AuNPs (Fig. 4a). The AuNPs particles become stable and protected by the citrate capping layer and can be stored under sterile conditions for several months.

Citrate capping of AuNPs could be replaced easily with various ligands such as peptides, proteins and oligonucleotides [39]. Thiol-linked oligonucleotides and peptides were employed to functionalize the AuNPs [40]. The AuNPs-FMDV biosensor for the conserved region of RNA dependent RNA polymerase of the FMDV genome (3D gene) was designed according to [41] with some modifications. Functionalization of the AuNPs with thiol-linked oligonucleotides and peptides are the most common approaches [40]. The prepared spherical 13 nm AuNPs have a maximum thiol-linked oligonucleotide loading density according to [33] who explored the relationship between the AuNPs size and the DNA loading density. The AuNPs-FMDV biosensor for the conserved region within the FMDV genome (3D gene) was designed according to [41] in which the thiol-linked polyA oligonucleotides for the 3D gene will replace the citrate ions and bind to the AuNPs. The thiol-linked poly(A) oligonucleotide length was 22 nucleotides for the forward primer and 17 nucleotides for reverse primer for the 3D gene with 10 poly(A) nucleotides with sequences as shown in (Table 1). This length ensured optimum immobilization according to [34]. Poly(A) nucleotides were used as a spacer for organized immobilization [33]. De-protection of the thiol-linked oligonucleotides (reduction by disulphide cleavage buffer) was conducted as previously reported [41]. After that, the purification of freshly reduced thiol-linked oligonucleotides was conducted by using the Nap-5 column, and the thiol-linked oligonucleotides were desalted and purified according to the manufacturer’s instructions. Design and characterization of the 3D AuNPs-FMDV biosensor and the conjugation process of the AuNPs and functionalization of AuNPs with poly A thiol-linked oligonucleotides was conducted following the published protocol [41] with some validation modification of the conjugation of the 400, 600 and 800 nM concentration of poly(A) thiol-linked oligonucleotides with 0.7 nM of 13 nm AuNPs.

To study the effect of the thiol-linked oligonucleotide concentration during the AuNPs-FMDV biosensor design, a standard curve was generated using standard RNA with the Real-time RT-PCR (rRT-PCR) to determine the limit of detection (LOD) and in regard to the successful real-time parameters as the cut-off CT value of FMDV lower than 32 as recommended by [42]; the acceptable CT difference value between 2 dilutions (slope) ranges between 3.1 and 3.58 and the acceptable percentage for the ability to conduct exponential amplification (efficiency %) is 90–110% [43]. In the present study, validation of the optimum thiol-linked oligonucleotide concentration, which has acceptable characteristics for the rRT-PCR assay, was tested with 400, 600 and 800 nM. The LOD, slope and efficiency % are illustrated in (Table 2). The thiol-linked oligonucleotide concentration of 400 nM showed acceptable success parameters with an efficiency of 94.5%, a slope − 3.544 and an LOD of 1 copy number as illustrated in Fig. 7a, b. Conversely, the thiol-linked oligonucleotide concentration of 600 and 800 nM had poor efficiency of 120 and 346% with a slope of − 2.8 and − 1.53; respectively, and with an LOD of 100 copy numbers for both as shown in Additional files 1, 2, and 3. Therefore, we recommend the conjugation with the 400 nM thiol-linked oligonucleotide concentration for designing the AuNPs-FMDV biosensor.

Moreover, to study the effect of the PCR conditions (the salt concentration in the PCR buffer and denaturation temperature), a comparison of optical densities of the AuNPs-FMDV biosensor before and after PCR was conducted. The peaks of the AuNPs after PCR have little variety from the peak intensity before PCR, but both have the same peak position at the 520 nm absorbance–wavelength; there was a slight disappearance of the immobilized thiol-linked oligonucleotide peak (260 nm) due to consumption of the primers in the PCR reaction (Fig. 5). This effect is observed is because the salt concentration in the PCR buffer was only three-tenths of that in Ref. [44]. Moreover, the denaturation temperature of 95 °C did not cause aggregation of the AuNPs because the sodium citrate cannot be reacted until the boiling temperature is achieved [13]. To study the stability of the AuNPs-FMDV biosensor, it was divided into aliquots and stored in a refrigerator at 4 °C for 6 months and each aliquot was tested by rRT-PCR to test the stability of the AuNPs-FMDV biosensor. The results revealed that the AuNPs-FMDV biosensor could be stored at 4 °C rather than − 20 °C for 6 months without changing its integrity and its analytical sensitivity.

**Fig. 6:**
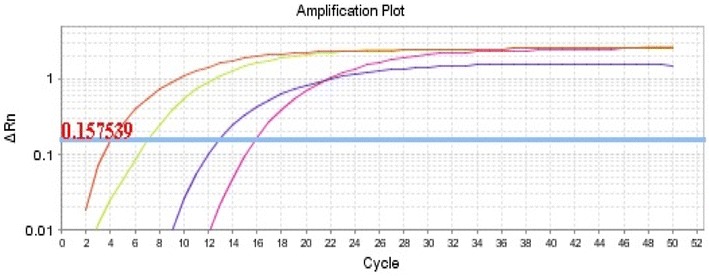
The amplification plot of FMDV isolates with classical rRT-PCR reagents and AuNPs-FMDV biosensor: the amplification plot is showing amplification of FMDV isolates with classical rRT-PCR reagents (yellow and violet) curves with CT values 7.03 and 15.9 and with AuNPs-FMDV biosensor (red and blue) curves with CT values 4.09 and 12.93

**Fig. 7:**
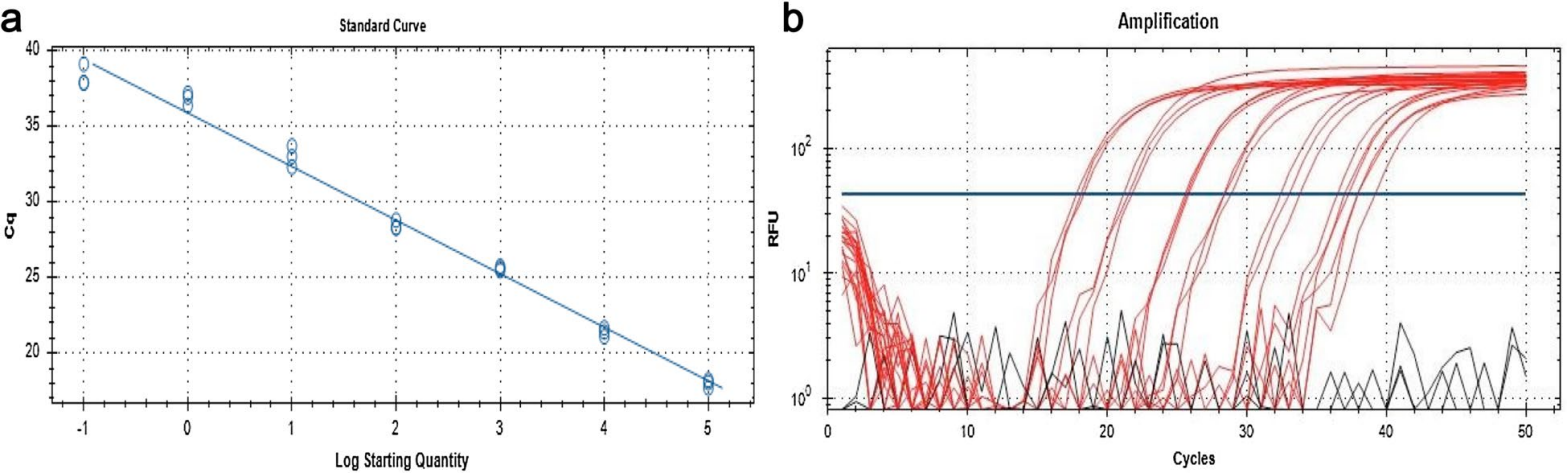
The standard and amplification curve of rRT-PCR assay using AuNPs-FMDV biosensor (400 nM primer): analytical sensitivity, dynamic range and limit of detection (LOD) of rRT-PCR assay using AuNPs-FMDV biosensor (400 nM primer) with serial dilution RNA standard of FMDV 3D gene show Eff. % = 94.5% and R^2^ = 0.989 and slope = − 3.544 standard curve (**a**) and amplification plot (**b**)

Harmonization and validation of the AuNPs 3D FMDV sensor with rRT-PCR and Real-time RT-PCR (rRT-PCR) for analytical sensitivity; generation of the standard curve using standard RNA was conducted on 3 replicates to determine the LOD regarding the success of the real-time parameters. The rRT-PCR reaction with the AuNPs-FMDV biosensor showed acceptable efficiency of 94.5%, slope − 3.544 and an LOD of 1 copy number (Fig. 6). The standard curve was generated using standard RNA with real-time RT-PCR (rRT-PCR) to determine the limit of detection (LOD) regarding the successful real-time parameters. The thiol-linked oligonucleotide concentration of 400 nM showed acceptable success parameters with an efficiency of 94.5%, a slope − 3.544 and an LOD of 1 copy number as illustrated in Fig. 7a, b. This result indicates that the AuNPs-FMDV biosensor increases the efficiency of the rRT-PCR and subsequently increases the yield of the PCR amplification with great attention to carrier animals and the detection of FMDV in the early stage before appearance of the clinical signs for successful prevention measures: treatment, vaccination and intensive culling. Similar to the previously reported results of [13], the AuNPs increased the yield of amplification five- to tenfold compared with that of classical PCR. Such an increase is due to the fundamental Nano effect of AuNPs and the appropriate immobilization of thiol-linked oligonucleotides on the AuNPs surface, which decrease the melting temperatures (Tm) of primers and therefore improve the PCR specificity in the annealing step via increasing the Tm difference between the complementary matched and mismatched primers [10]. Likewise, they also result in unique optical properties, good stability, excellent biocompatibility, simplicity in preparation and easy surface modification, and the AuNPs have multiple effects on Nano PCR. Several studies on such effects explain the mechanism of enhancement, including the following: (I) AuNPs adsorb polymerase and modulate the amount of active polymerase in PCR. (II) AuNPs adsorb PCR products and improve PCR efficiency via increasing the dissociation of the PCR products in the denaturing step [10]. (III) There is an exceptional efficiency in the heat dispersion of AuNPs in shortened PCR cycles [13]. (IV) AuNPs modulate the activity of the DNA polymerases and achieve a hot start, similar to PCR with conventional *Pyrococcus furiosus* (Pfu) polymerase [14]. Several potential mechanisms for the effects of AuNPs have been proposed including; (a) selective binding of single-stranded DNA (ssDNA) to nanomaterials in a manner analogous to the ssDNA-binding protein (SSBs) [14, 45], (b) the excellent heat transfer property of the nanomaterials [13], (c) adsorption of DNA polymerases with nanomaterials [14, 46], (d) condensation of PCR reactants on the surface of the nanomaterials [47], (e) the catalytic property of the nanomaterials [48, 49]. With real-time RT-PCR (rRT-PCR) for the diagnostic sensitivity and specificity, the FMDV and SVDV isolates were tested with classical rRT-PCR reagents and with the AuNPs-FMDV. As expected, the AuNPs-FMDV biosensor can increase the sensitivity of rRT-PCR and the detection limit by more than tenfold [13], the obtained CT values were different in the rRT-PCR with an AuNPs-FMDV biosensor compared to without the biosensor (a CT of 4.09 and 12.9) and (7.03 and 15.9), respectively, and no cross reactivity was observed with SVDV isolates as NCT as shown in (Fig. 6).

**Fig. 8:**
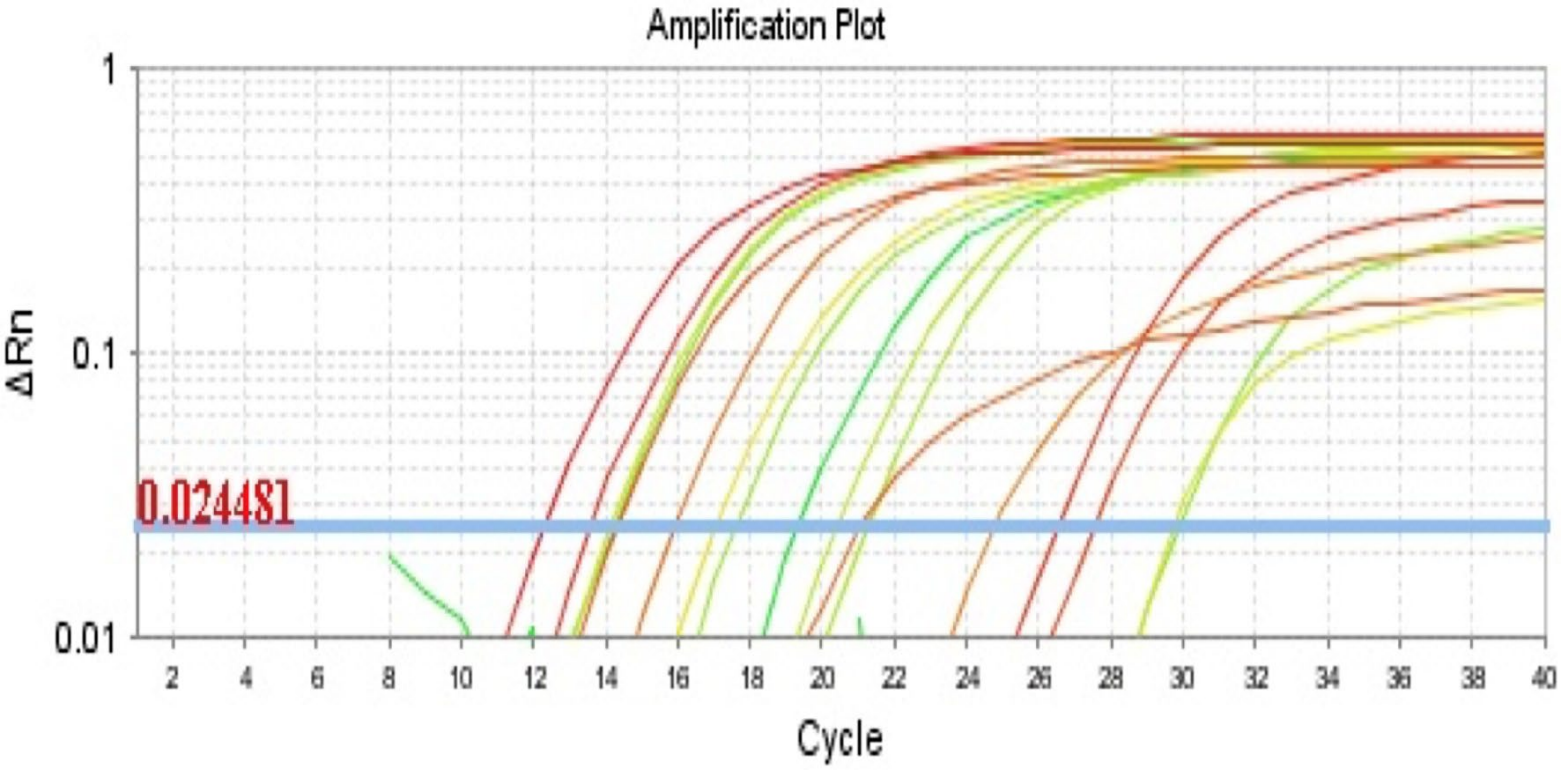
The amplification plot with CT values of the field samples with AuNPs-FMDV biosensor: Red curves represent field samples with AuNPs-FMDV biosensor with CT value 12.29, 13.54, 26.48, 27.44, 14.26, 21.02, 15.89, 24.74. Green curves represent field samples with classical rRT-PCT reagent with CT values 14.1, 17, 29.66, 29.82, 19.32, 24.03, 17.6, 26.82

Application of the developed AuNPs-FMDV biosensor with rRT-PCR was tested with 31 clinical samples (unruptured and recently ruptured vesicles in the buccal cavity, vesicular fluid, epithelium and hearts), which were collected from cattle, buffalo and calves from Egypt during March 2012 to September 2015. The AuNPs-FMDV biosensor demonstrated an enhancement in sensitivity and the amplification plot (Fig. 8) showed a tenfold increase in LOD, whereas a large difference in the CT value of at least 3–3.5 was observed (Table 3). The enhancement of the PCR efficiency using the AuNPs-FMDV biosensor depends on the nature of the clinical samples used [13]. Amplification and multicomponent plots with CT values of the field samples; the red curve represents field samples with the AuNPs-FMDV sensor with CT values of 12.29, 13.54, 26.48, 27.44, 14.26, 21.02, 15.89, and 24.74 and the green curve represents field samples with classical rRT-PCT reagent with CT values of 14.1, 17, 29.66, 29.82, 19.32, 24.03, 17.6, and 26.82 (Fig. 8). Amplification and multicomponent plots of the swine vesicular disease virus (SVDV) as a negative control with the AuNPs-FMDV sensor and with the classical rRT-PCT reagent (Fig. 8).

## Conclusion

The AuNPs-FMDV biosensor demonstrates a superior analytical and clinical performance for the FMDV diagnosis. The analytical sensitivity and dynamic range LOD of the AuNPs-FMDV biosensor was 10 copy numbers of the RNA standard in RT-PCR and 1 copy number of the RNA standard in the rRT-PCR with a 94.5% efficiency, 0.989 R^2^, a − 3.544 slope and 100% specificity without cross reactivity with SVDV. It has a simple workflow and it accelerates epidemiological surveillance. Hence, it is suitable for quarantine stations and farms for diagnosis, particularly in FMDV endemic areas.

## Additional files

**Additional file 1.** Ethidium bromide stained agarose electrophoresis containing classical and AuNPs-FMDV biosensor PCR product: ethidium bromide stained agarose electrophoresis containing the PCR product of 3D gene with 106 base pairs (bp) with classical PCR reaction (C) and modified PCR reaction with AuNPs-FMDV biosensor (S) using standard RNA dilution from Log 5 till Log 0 with Swine Vesicular Disease Virus (SVDV) as a negative control (−ve) with 50 bp molecular marker. The figure illustrated that Classical PCR reaction could detect till Log 3 (C3), moreover it had nonspecific bands, but modified PCR reaction with AuNPs-FMDV biosensor (S) could detect till 100 copies number with more band intensity and without nonspecific bands with no cross reactivity with SVDV.

**Additional file 2.** The standard curve of AuNPs-FMD biosensor (0.7 nM AuNPs and 600 nM Primer): analytical sensitivity, dynamic range and limit of detection (LOD) of rRT-PCR assay using AuNPs-FMDV biosensor (600 nM thiol-linked oligonucleotides) with serial dilution RNA standard of FMDV 3D gene. The figure illustrated Standard Curve AuNPs-FMDV biosensor with serial dilution RNA standard of FMDV 3D gene with Eff. % = 120% and slope = − 2.8.

**Additional file 3.** The standard curve of AuNPs-FMD biosensor (0.7 nM AuNPs and 800 nM Primer): analytical sensitivity, dynamic range and limit of detection (LOD) of rRT-PCR assay using AuNPs-FMDV biosensor (800 nM thiol-linked oligonucleotides) with serial dilution RNA standard of FMDV 3D gene. The figure illustrated standard curve AuNPs-FMDV biosensor with serial dilution RNA standard of FMDV 3D gene with Eff. % = 346% and slope = − 1.53
