# Supplementary figures and images for: Development of gold nanoparticles biosensor for ultrasensitive diagnosis of foot and mouth disease virus
Source: J Nanobiotechnology. 2018 May 11;16:48. doi: 10.1186/s12951-018-0374-x (PMC5946443; doi:10.1186/s12951-018-0374-x)

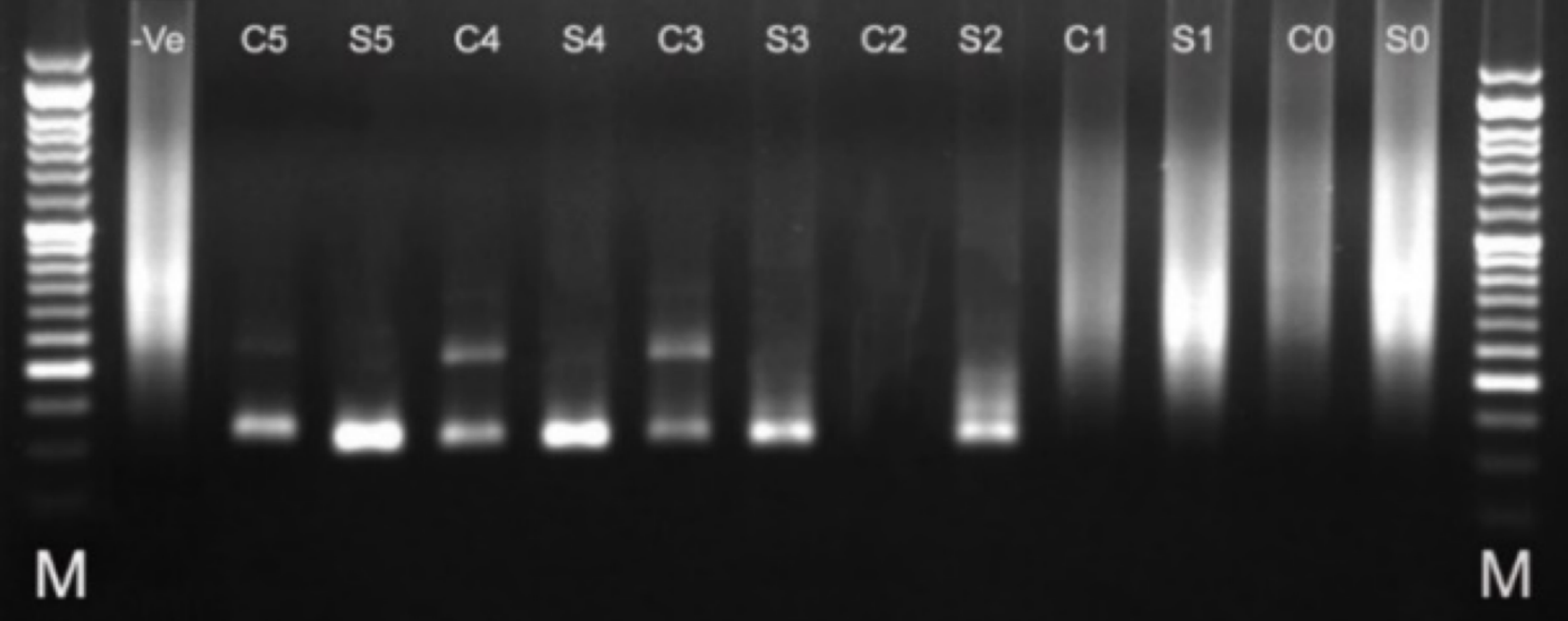

Supplement: Supplementary file 1 — Additional file 1. Ethidium bromide stained agarose electrophoresis containing classical and AuNPs-FMDV biosensor PCR product: ethidium bromide stained agarose electrophoresis containing the PCR product of 3D gene with 106 base pairs (bp) with classical PCR reaction (C) and modified PCR reaction with AuNPs-FMDV biosensor (S) using standard RNA dilution from Log 5 till Log 0 with Swine Vesicular Disease Virus (SVDV) as a negative control (−ve) with 50 bp molecular marker. The figure illustrated that Classical PCR reaction could detect till Log 3 (C3), moreover it had nonspecific bands, but modified PCR reaction with AuNPs-FMDV biosensor (S) could detect till 100 copies number with more band intensity and without nonspecific bands with no cross reactivity with SVDV. [file 12951_2018_374_MOESM1_ESM.pdf]

# Standard Curve

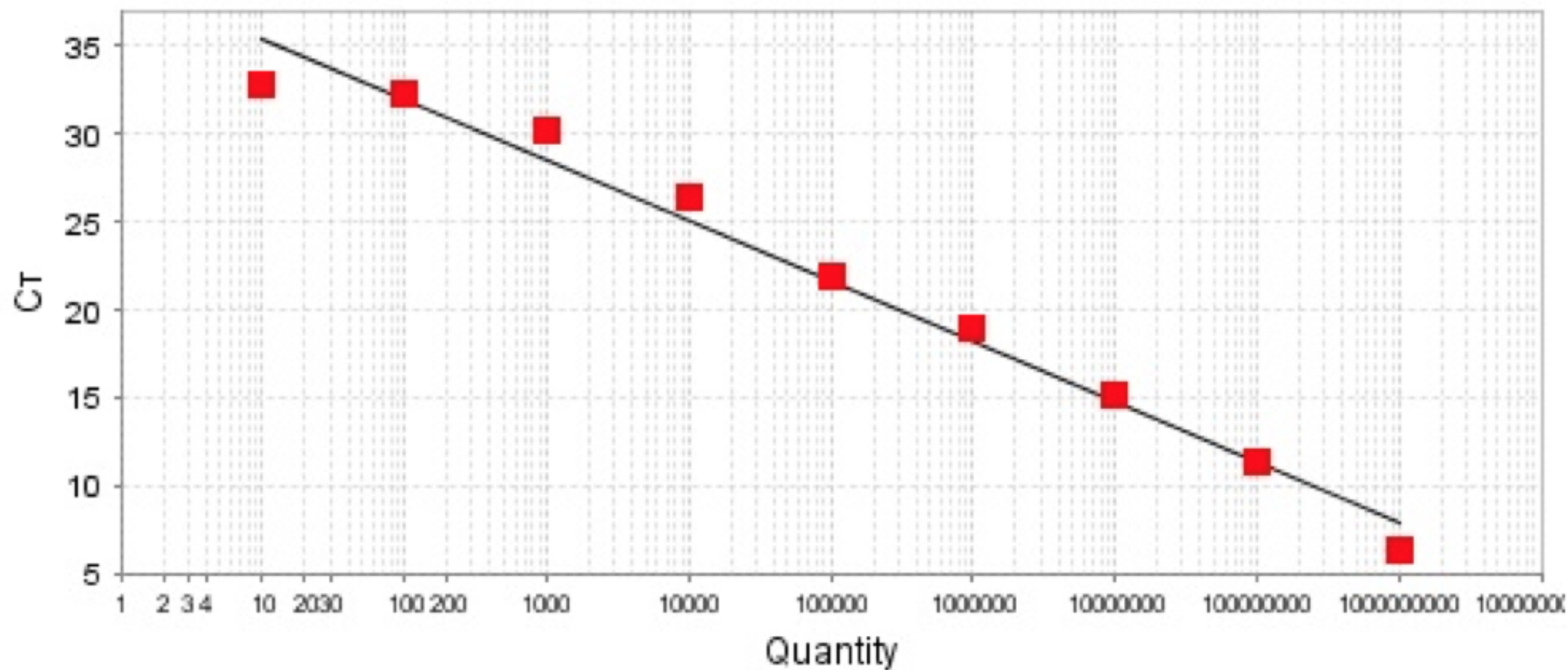

Supplement: Supplementary file 2 — Additional file 2. The standard curve of AuNPs-FMD biosensor (0.7 nM AuNPs and 600 nM Primer): analytical sensitivity, dynamic range and limit of detection (LOD) of rRT-PCR assay using AuNPs-FMDV biosensor (600 nM thiol-linked oligonucleotides) with serial dilution RNA standard of FMDV 3D gene. The figure illustrated Standard Curve AuNPs-FMDV biosensor with serial dilution RNA standard of FMDV 3D gene with Eff. % = 120% and slope = − 2.8. [file 12951_2018_374_MOESM2_ESM.pdf]

# Standard Curve

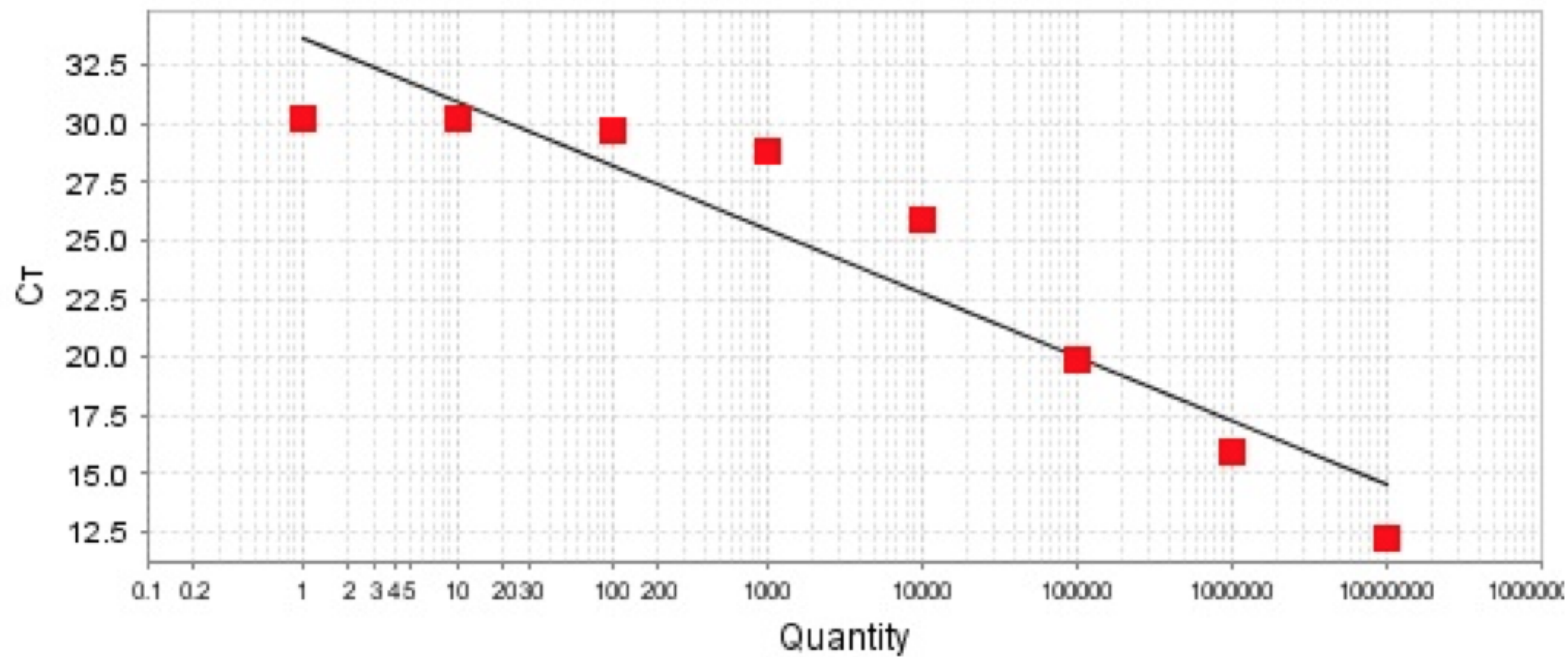

Supplement: Supplementary file 3 — Additional file 3. The standard curve of AuNPs-FMD biosensor (0.7 nM AuNPs and 800 nM Primer): analytical sensitivity, dynamic range and limit of detection (LOD) of rRT-PCR assay using AuNPs-FMDV biosensor (800 nM thiol-linked oligonucleotides) with serial dilution RNA standard of FMDV 3D gene. The figure illustrated standard curve AuNPs-FMDV biosensor with serial dilution RNA standard of FMDV 3D gene with Eff. % = 346% and slope = − 1.53 [file 12951_2018_374_MOESM3_ESM.pdf]
